# E1-Like Activating Enzyme Atg7 Is Preferentially Sequestered into p62 Aggregates via Its Interaction with LC3-I

**DOI:** 10.1371/journal.pone.0073229

**Published:** 2013-09-04

**Authors:** Wentao Gao, Zhixia Chen, Wei Wang, Michael T. Stang

**Affiliations:** 1 Department of Surgery, University of Pittsburgh School of Medicine, Pittsburgh, Pennsylvania, United States of America; 2 College of Animal Science and Veterinary Medicine, Jilin University, Changchun, Jilin, China; Peking University Health Science Center, China

## Abstract

p62 is constitutively degraded by autophagy via its interaction with LC3. However, the interaction of p62 with LC3 species in the context of the LC3 lipidation process is not specified. Further, the p62-mediated protein aggregation’s effect on autophagy is unclear. We systemically analyzed the interactions of p62 with all known Atg proteins involved in LC3 lipidation. We find that p62 does not interact with LC3 at the stages when it is being processed by Atg4B or when it is complexed or conjugated with Atg3. p62 does interact with LC3-I and LC3-I:Atg7 complex and is preferentially recruited by LC3-II species under autophagic stimulation. Given that Atg4B, Atg3 and LC3-Atg3 are indispensable for LC3-II conversion, our study reveals a protective mechanism for Atg4B, Atg3 and LC3-Atg3 conjugate from being inappropriately sequestered into p62 aggregates. Our findings imply that p62 could potentially impair autophagy by negatively affecting LC3 lipidation and contribute to the development of protein aggregate diseases.

## Introduction

Macroautophagy (hereinafter referred to as autophagy) is a highly conserved cellular degradation process for senescent proteins and damaged organelles [1], [2]. It is a process of cellular membrane trafficking characterized by a double membrane bound autophagosome engulfing intracellular targets [3], [4]. The autophagosome delivers its contents by fusion with the lysosome for the end point of degradation [5].

In contrast to the ubiquitin-proteasome degradation system, there are two ubiquitin-like conjugation pathways essential for autophagosome formation: Atg12-Atg5 and Atg8/LC3-phosphatidylethanolamine (LC3-PE) conjugation processes [6], [7]. In the Atg12 conjugation system, Atg12 forms an intermediate with an E1-like activating enzyme Atg7 via its C-terminal glycine residue. The Atg12-Atg7 conjugate then exchanges with an E2-like enzyme Atg10 to form an Atg12-Atg10 conjugate as Atg12 is transferred to its distal target Atg5 to form a stable Atg12-Atg5. Finally, Atg12-Atg5 proceeds downstream to interact with Atg16L1 to form an Atg16L1:Atg5–Atg12 complex. The Atg8/LC3-PE lipidation process involves a pro-LC3 being cleaved by a cysteine protease Atg4B to expose the C-terminal glycine reside resulting in LC3-I. Three conjugates or intermediate species are sequentially generated in the pathway of LC3-I to LC3-PE lipidation. LC3-I is activated by Atg7 as a thioester bond is formed between a catalytic cysteine residue of Atg7 and the C-terminal glycine of LC3-I. The LC3-Atg7 intermediate exchanges with Atg3, an E2-like enzyme, in forming a second thioester intermediate LC3-Atg3 [8]. Finally, the lipidation process is completed by the putative E3-like enzyme Atg16L1:Atg5–Atg12 complex as LC3-Atg3 is exchanged with PE to form LC3-II [9].

Sequestosome 1/p62 (hereinafter referred to as p62) is a scaffold protein involved in disparate signaling pathways. It binds the tyrosine kinase Lck and atypical protein kinase C [10], [11]. It functions as an adaptor protein in NFκB signaling pathways activated by tumor necrosis factor-alpha [12]. p62 has also been described to be involved in the activation of caspase-8 upon stimulation of cell death receptors [13]. It is required for Ras-induced tumorigenesis and is clearly up-regulated in different human tumors and correlates with aggressive progression of prostate and breast cancers [14]. Moreover, p62 participates in protein aggregate formation observed as ubiquitin-related inclusions in hepatic injury and various neurodegenerative diseases (e.g. alcoholic hepatitis, steatohepatitis, Huntington disease, Parkinson disease, amyotrophic lateral sclerosis) [15], [16], [17]. p62 is degraded by proteasome via ubiquitination and by autophagy via interaction with LC3, respectively [18], [19].

In this study, we sought to systemically analyze p62 interactions with LC3 species in the LC3-PE conjugation process to understand its effect on autophagy process. We find that p62 interacts with LC3-I and Atg7:LC3-I complex but not Atg4B and Atg3. Given that p62 is a major component within pathologic protein aggregates, our findings may have clinical significance in understanding the role of p62 in autophagy and related protein aggregate diseases.

## Materials and Methods

### Reagents and Antibodies

Cell culture reagents were purchased from LONZA (Walkersville, MD). The following antibodies were used: rabbit polyclonal anti-LC3B, Atg4B, Atg3, and Atg7 antibodies (Cell Signaling); mouse monoclonal anti-GFP and p62 antibodies (Santa Cruz biotech); mouse monoclonal anti-Flag M2 and β-actin antibodies (Sigma); GFP-Trap (Allele Biotechnology); anti-rabbit IgG (Alexa Fluor 488 conjugate) and anti-mouse IgG (Alexa Fluor 555 conjugate) (Cell signaling); anti-mouse IgG(AMCA-conjugate) (Jackson ImmunoResearch). All other reagents were purchased from Sigma.

### Cell Culture and Stable Cell Lines

HEK293 cell (ATCC), A549 cell (ATCC), MEFwt, and MEFatg5^−/−^ cells (provided by Dr. Noboru Mizushima), and have been described [20]. Cells were grown in DMEM media supplemented with 10% fetal bovine serum, 2 mM L-glutamine, and 100 U/ml penicillin/streptomycin in a 5% CO2 incubator at 37°C. Stable lines were made by infection of cells with a retroviral vector expressing GFP fusion with human LC3B gene (HEK293-GFP-LC3) or expression plasmid (HEK293-GFP-LC3^A120^) and selected in DMEM media with 800 µg/ml of G418.

### Atg Gene Expression Vector Construction

Human Atg4B, Atg7, Atg3 and Atg12 Open Reading Frame (ORF) were cloned into the pEGFP-C1 expression vector (Clonetech). Point mutations were generated by site-directed mutagenesis on GFP-Atg4B, GFP-Atg7, and GFP-Atg3 expression vectors, respectively. Human p62 ORF was cloned into pEGFP-C1 and pmRFP-C1 (Clonetech). N-terminal 3×Flag tag fused human LC3B ORF (Flag-LC3) was cloned into pAdlox vector [21]. Flag-LC3^A120^ mutant was generated by removing the coding sequence of the last five amino acids from wild-type human LC3B ORF and replacing glycine 120 with Alanine. Similarly, GFP-LC3 and GFP-LC3^A120^ were cloned into the pAdlox vector. All inserted sequences were validated by DNA sequencing.

### Immunofluorescence Staining of Cells and Florescence Microscopy

Transfected cells were cultured for a defined time period, fixed in 4% paraformaldehye/1×PBS for 15 min, and washed three times with 1 × PBS; images were then recorded using EVOSfl microscopy. For immunofluorescence staining of cells, cells were cultured on coverslips overnight and transfected with indicated plasmids or treated with CPP. Cells were then fixed with 4% paraformaldehyde/1 × PBS for 15 min. Cells were washed three times with 1 × PBS and permeabilized using 0.1% Triton X-100 in 1 × PBS, and blocked with 5% of BSA and sequentially administered primary antibody and secondary antibody. Stained cells were examined and recorded using fluorescence microscopy.

### Immunoprecipitation and Western Blot Analysis

HEK293 cells were transfected with plasmids and cultured for 48 hours. Cells were rinsed with ice-cold 1 × PBS, scraped, collected by centrifugation at 4°C, and lysed in cell RIPA lysis buffer (Cell Signaling) with protease inhibitor cocktails (Sigma). Lysates were centrifuged at 15,000 *g* for 15 min at 4°C and the supernatants were collected. Immunoprecipitation (IP) was performed in native conditions using Flag M2 antibody, p62 antibody or GFP-trap at 4°C. 10 µg or 20 µg of total cell lysates were used as non IP control. Protein samples were separated by a 12% SDS-PAGE and transferred to Reinforced NC membrane (Whatman GmbH). The membranes were blocked with 5% skim milk in 0.1% Tween 20/1 × PBS and incubated with primary antibodies. Blots were probed with horseradish peroxidase (HRP)-conjugated anti-mouse or anti-rabbit IgG (Jackson ImmunoResearch). Bands were then visualized using SuperSignal West Pico or West Femto Chemiluminescent Substrate (Thermo Scientific). For p62 effect on LC3lipidation, adenoviral vector encoding mRFP-p62 was used to infect A549 cell at dose of 20 MOI (D1) and 10 MOI (D2). 24 hours later, cells were starved in Earle’s balanced salt solution (EBSS) for 2 h and total cell lysates were used for Western blot analysis.

## Results

### p62 does not Sequester Atg4B within p62 Aggregates

**Figure 1:**
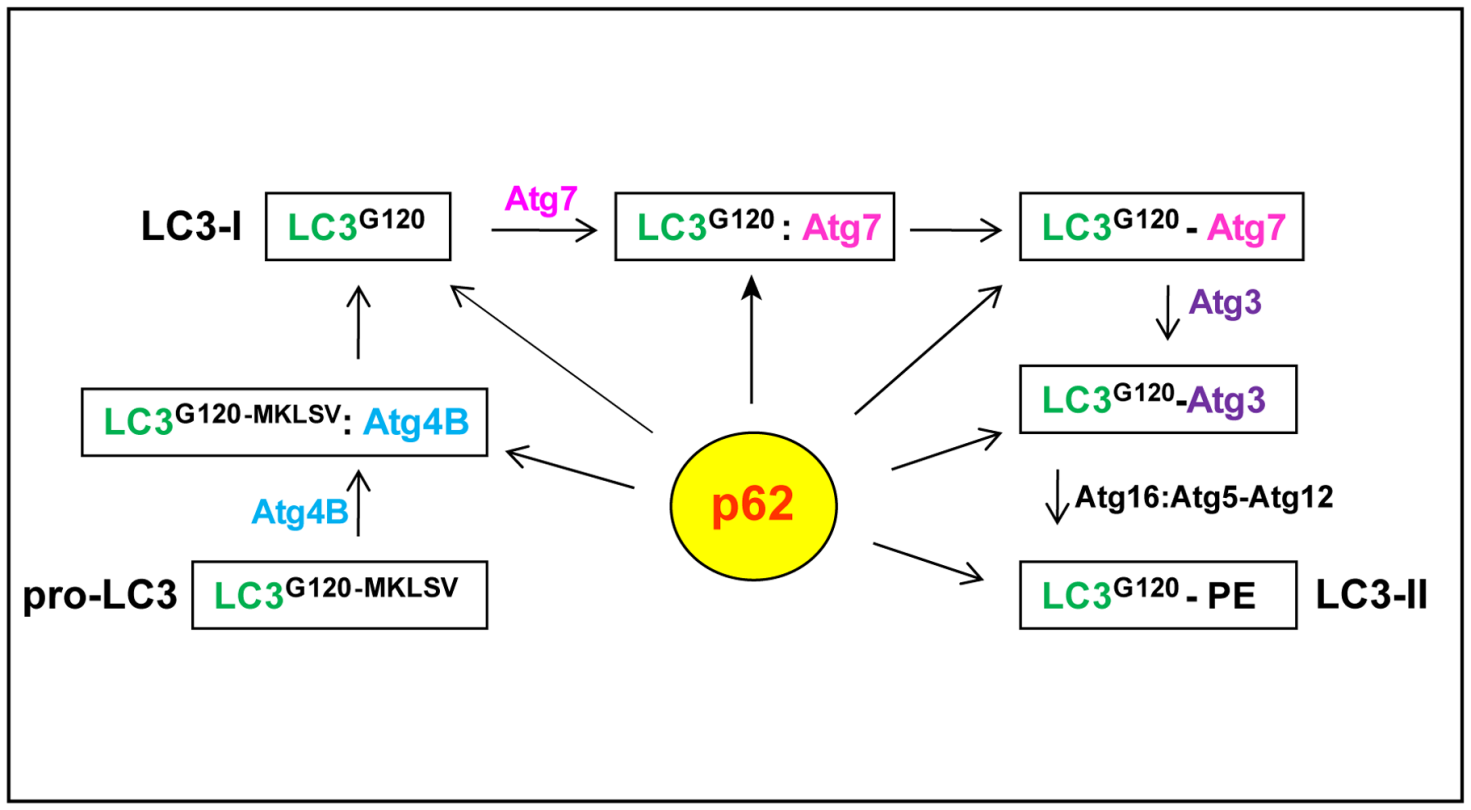
Potential Interaction stages of p62 with LC3 species in the course of LC3 lipidation. The known stages for LC3 lipidation are boxed. After translation pro-LC3B is processed by Atg4B by removing the last five amino acids to expose the G120 residue (LC3-I). A portion of LC3-I forms a complex with the E1-like enzyme Atg7 and subsequently generates a LC3-Atg7 conjugate. LC3-Atg7 then interacts with the E2-like enzyme Atg3 to form a LC3-Atg3 conjugate which interacts with Atg16L1:Atg5-Atg12 complex to convert to LC3-II under autophagic stimulation. p62 could potentially bind any LC3 specie at any stage of this process.

Figure 1 shows the known reactions in the LC3 lipidation process. We used human LC3B (referred to as LC3) as a model to address p62 and LC3 interactions. We aimed to define at which stage(s) p62 could interact with LC3 during the course of LC3 lipidation and to understand the role of p62 protein aggregates in the autophagy process.

**Figure 2:**
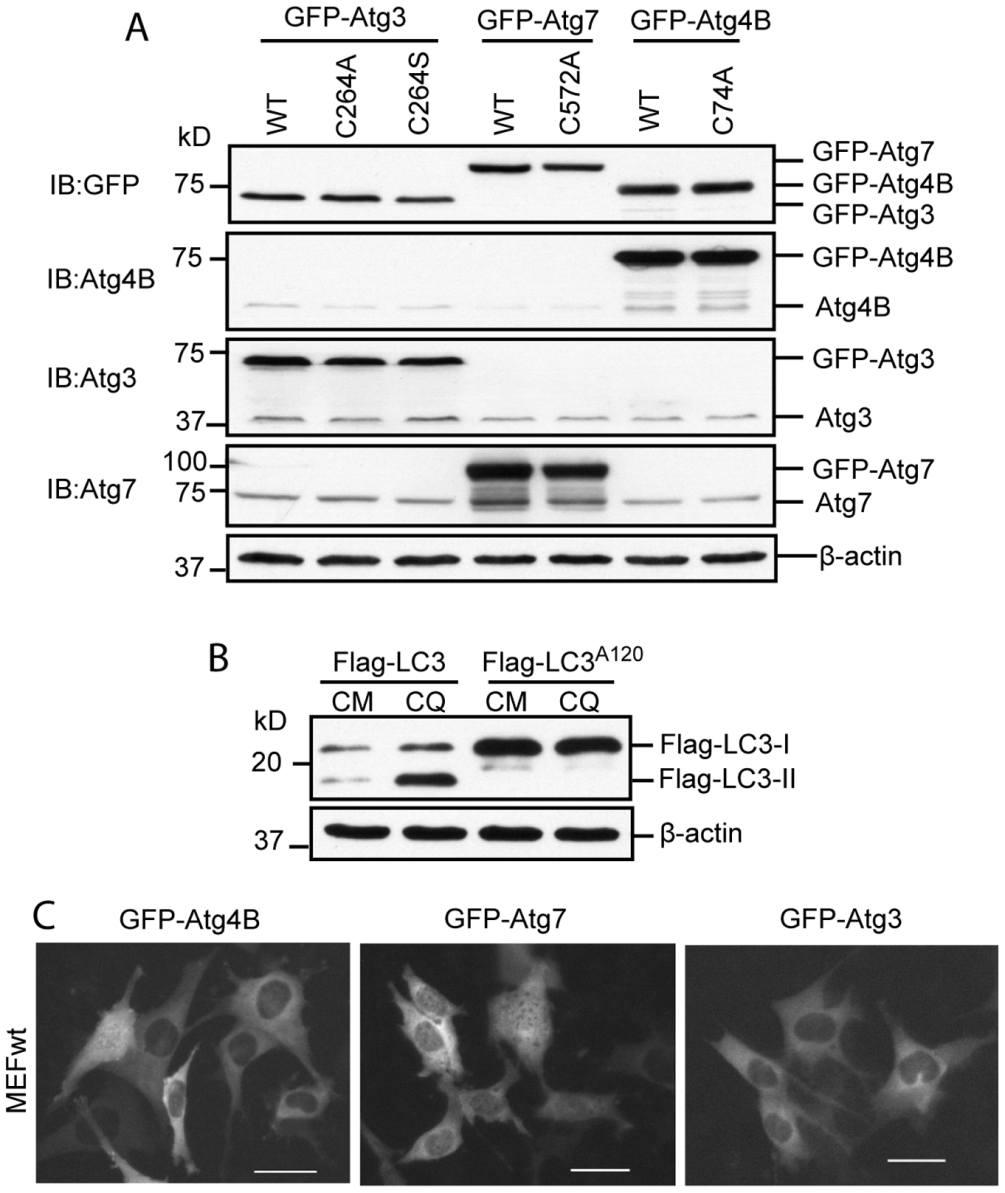
Validation of recombinant Atg gene expression. (A) Expression plasmids were transfected into HEK293 cells for 24 h. Cell lysates were used to detect the fusion genes’ expression by Western blot using antibodies as indicated. (B) Flag-LC3 or Flag-LC3^A120^ was transfected into HEK293 cells for 24 h. 50 µM of Chloroquine (CQ) was used to induce Flag- LC3-II accumulation for 2 h before harvesting the cells. Flag-LC3 is normally lipidated as demonstrated by Flag-LC3-II accumulation after CQ treatment. No such accumulation was seen in Flag-LC3^A120^ mutant after CQ treatment demonstrating that this mutant does not pass through the lipidation process. (C) MEFwt cells were transfected with indicated expression plasmids for 24 h before they were recorded by fluorescence microscopy. GFP-Atg4B, GFP-Atg7 and GFP-Atg3 were mainly distributed in the cytosol. No puncta was observed at any given time. Scale bar = 25micron.

Expression of GFP tagged Atg4B, Atg7, Atg3 and their inactivated mutants, Flag tagged LC3 and LC3^A120^ were validated in HEK293 cells (Fig. 2, A and B). GFP-Atg4B, GFP-Atg7 and GFP-Atg3 were distributed within the cytosol. No punctae were observed at any given time (Fig. 2, C).

Self-oligomerization of transient expression of p62 promotes protein aggregate formation which is observed as punctae. We used fluorescence protein tagged p62 as a marker to analyze p62 and LC3 interaction in cells. Should LC3 species interact with p62, they would be incorporated into p62 punctae. As such, we can observe these interactions in cells utilizing fluorescence microscopy.

**Figure 3:**
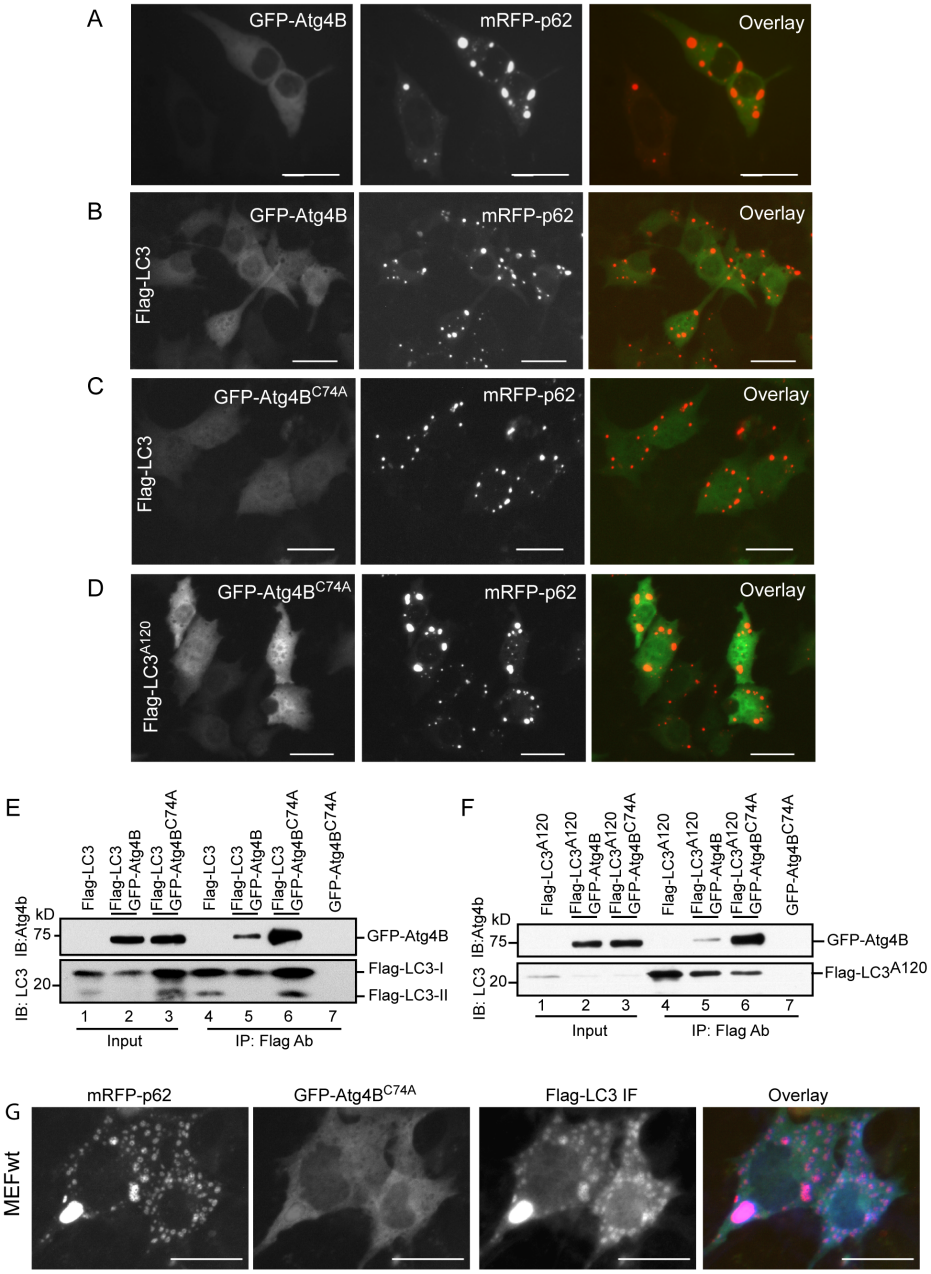
p62 aggregates do not sequester Atg4B. GFP-Atg4B and mRFP-p62 expression plasmids (A) or with Flag-LC3 (B) were co-transfected into MEFwt cells for 24h. GFP-Atg4B^C74A^ and mRFP-p62 with Flag-LC3 vectors (C) or with Flag-LC3^A120^ vector (D) were co-transfected into MEFwt cells for 24 h. GFP-Atg4B or GFP-Atg4B^C74A^ expression vector was co-transfected with Flag-LC3 (E) or Flag-LC3^A120^ (F) into HEK293 cells for 48 h. Immunoprecipitation (IP) was performed using total cell lysates. Immunoblotting was conducted using the antibodies as indicated. (G) mRFP-p62, GFP-Atg4B^C74A^ and Flag-LC3 expression vectors were co-transfected into MEFwt cells for 24 h. Colocalization of Flag-LC3 within mRFP-p62 aggregates were detected by immunofluorescence staining (IF) using Flag M2 antibody. Fluorescence images presented in this study were representative of at least three independent experiments. Scale bar = 25micron.

To accomplish cleavage of pro-LC3, a protein complex must form between a substrate (pro-LC3) and enzyme (Atg4B). At this stage, pro-LC3:Atg4B complex may interact with p62 and be sequestered within p62 aggregates. No GFP-Atg4B was observed in mRFP-p62 puncta by co-expression of mRFP-p62 and GFP-Atg4B (Fig. 3A). To increase the amount of LC3:Atg4B complex and augment the likelihood of LC3:Atg4B complex interaction with p62, mRFP-p62/GFP-Atg4B/Flag-LC3 were co-expressed. However, this manipulation did not result in the colocalization of Atg4B in p62 puncta (Fig. 3B).

Atg4B^C74A^ mutant has been previously shown to confer strong binding to LC3 [22]. Co-expression of mRFP-p62, Flag-LC3 and GFP-Atg4B^C74A^ did not lead to GFP-Atg4B^C74A^ being included into mRFP-p62 puncta either (Fig. 3C). Similar results were found in co-expression of mRFP-p62, Flag-LC3^A120^ and GFP-Atg4B^C74A^ (Fig. 3D).

To confirm the interactions between LC3 and Atg4B, we performed immunoprecipitation assays by co-expressing GFP-Atg4B or GFP-Atg4B^C74A^ with Flag-LC3 or Flag-LC3^A120^ in HEK 293 cells. Both wild-type GFP-Atg4B and GFP- Atg4B^C74A^ mutant were pulled down by Flag-LC3 or Flag-LC3^A120^ (Fig. 3E and F). Of note, a greater amount of Atg4B^C74A^ was observed in this context indicating that Atg4B^C74A^ interaction with LC3 or LC3^A120^ is indeed significantly enhanced (Fig. 3E, lane 6 and F, lane 6). Further, co-expression of mRFP-p62/GFP-Atg4B^C74A^/Flag-LC3 demonstrated Flag-LC3 was indeed sequestered in mRFP-p62 puncta while no p62 puncta was positive for GFP-Atg4B^C74A^ (Fig. 3G).

Together, these results demonstrate that Atg4B does not interact with p62, and once bound to Atg4B, pro-LC3 or LC3-I lost its ability to interact with p62. Thus, Atg4B and Atg4B bound LC3 are protected from being sequestered in p62 aggregates.

### LC3-I is Sequestered within p62 Aggregates

**Figure 4:**
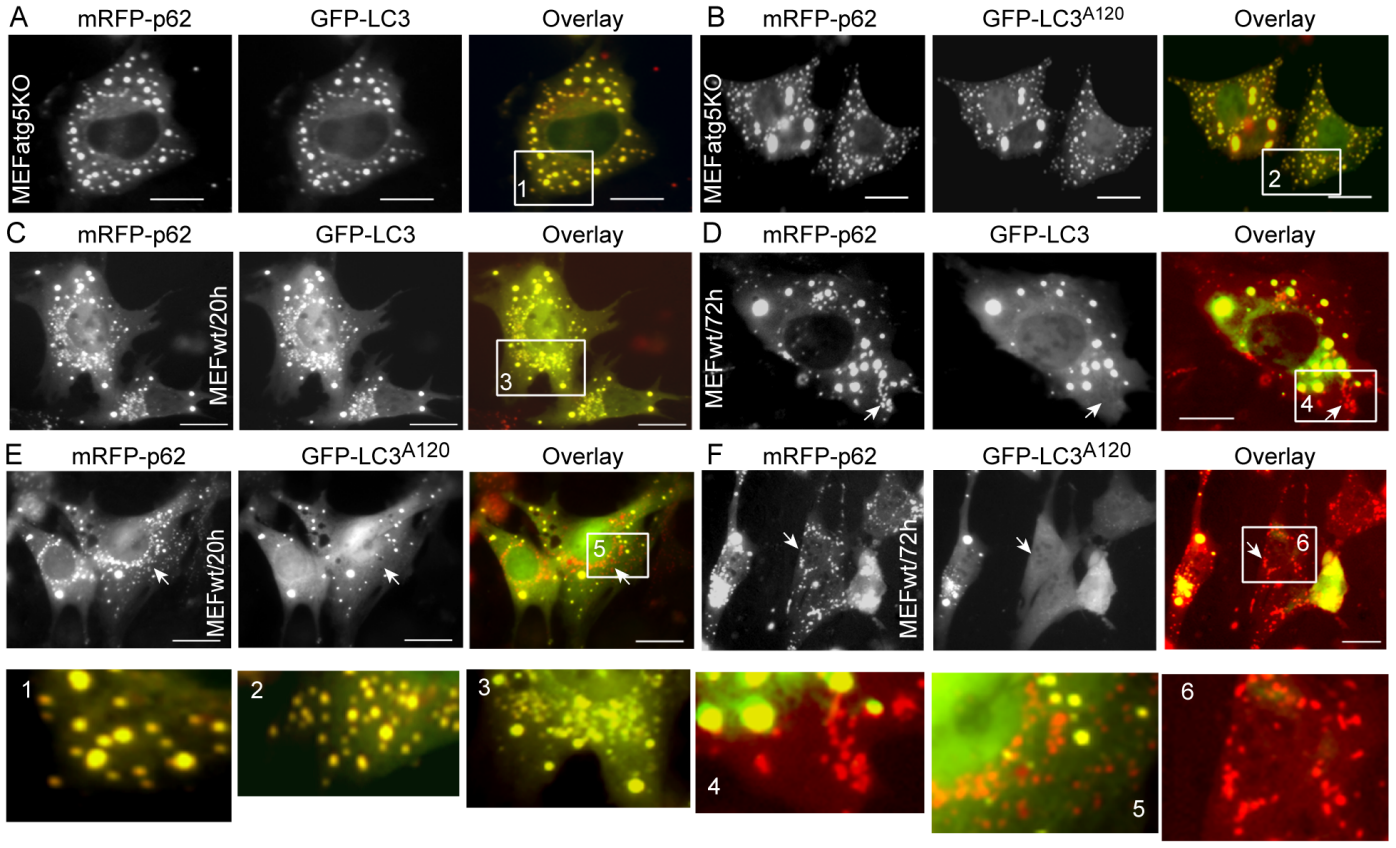
p62 aggregates sequester LC3-I. Vectors expressing mRFP-p62, GFP-LC3, or GFP-LC3^A120^ were transfected into MEFatg5^−/−^ cells as indicated (A and B). Images were taken at 48 h time point. The same sets of plasmids were transfected into MEFwt cells as indicated for 72 h. Images were recorded at 20 h and 72 h post transfection (C–F). Scale bar = 25micro. Arrows point to mRFP-p62 only punctae. The inserts were enlarged to show the puncta in detail.

After processed by Atg4B, pro-LC3 exposes its C-terminal glycine residue and turns to LC3-I (Fig. 1). LC3-I is the predominant LC3 species compared to other LC3 species in the LC3 lipidation process. As such, LC3-I can be considered a “stock” LC3 species for downstream series reactions with Atg7, Atg3 and PE. Considering the absence of LC3-II generation in MEFatg5^−/−^ cells, utilizing such a model excludes the possibility of p62 interaction with LC3-II. The GFP-LC3^A120^ mutant is equivalent to GFP-LC3-I except that it cannot form a conjugate with Atg7. We employed this mutant to address the interaction between p62 and LC3-I. All mRFP-p62 punctae were positive for GFP-LC3 or GFP-LC3^A120^ at all observation time points in MEFatg5^−/−^ cells (Fig. 4A and B). In contrast, nearly all mRFP-p62 punctae were positive for GFP-LC3 in MEFwt cells at the 20 hour time point (Fig. 4C). At 72 hours, mRFP-p62 positive but GFP-LC3 negative punctae were observed (Fig. 4D). This indicates that at earlier time points mRFP-p62 punctae likely represent p62 aggregates or autophagic vacuoles in MEFwt cells. Over time, some of those p62 aggregates were delivered to the lysosome compartment where GFP-LC3 fluorescence was quenched but mRFP-p62 signal persists at the later time point [23]. Comparatively, mRFP-p62 positive but GFP-LC3^A120^ negative punctae were predominant and observed in MEFwt cells at all time points (Fig. 4 E and F). Of note, larger mRFP-p62 punctae were also positive for GFP-LC3^A120^ in MEFwt cells, indicating that p62 could still interact with GFP-LC3^A120^ in wild-type cells but with less efficiency.

Altogether, these results demonstrate that p62 efficiently binds and sequesters LC3-I within p62 aggregates in autophagy deficient cells, which likely occurs via the physical interactions between these two molecules. However, p62 preferentially binds LC3 species other than LC3-I in wild-type cells, implying that p62 may be degraded by autophagy via its interaction with LC3-II in wild-type cells.

### Atg7 is Sequestered within p62 Aggregates in a LC3-Dependent Manner

**Figure 5:**
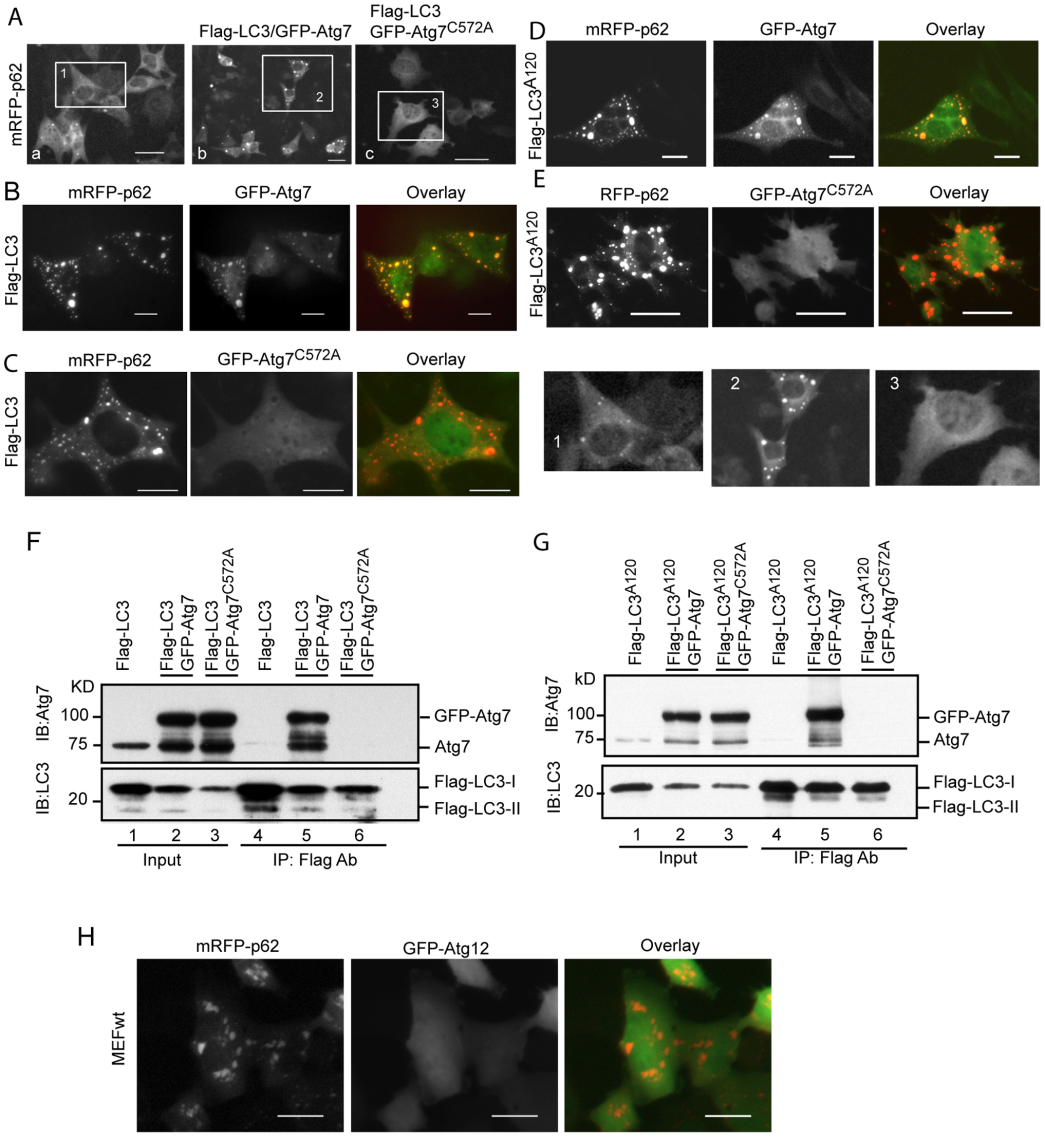
p62 aggregates sequester Atg7 in a LC3-dependent manner. GFP-Atg7 (Aa), GFP-Atg7/Flag-LC3 (Ab), or GFP-Atg7^C572A^/Flag-LC3 (Ac) vectors were co-transfected with mRFP-p62 into MEFwt cells for 48 h. GFP-Atg7 punctae were shown; mRFP-p62 and Flag-LC3 with GFP-Atg7 vectors (B) or with GFP-Atg7^C572A^(C) were co-transfected into MEFwt cells for 48 h; mRFP-p62 and Flag-LC3A^120^ with GFP-Atg7 vectors (D) or with GFP-Atg7^C572A^ (E) were co-transfected into MEFwt cells for 48 h. Flag-LC3, GFP-Atg7, or GFP-Atg7^C572A^ expression vectors were co-transfected into HEK293 cells as indicated for 48 h. Total cell lysates were used for IP by the Flag M2 antibody. Immunoblotting was conducted using the antibodies as indicated (F and G). Expressing vectors of GFP-Atg12 and mRFP-p62 were co-transfected into MEFwt cells for 48 h (H). All images were recorded at 48 h post-transfection. Scale bar = 25 micron. The inserts were enlarged to show the puncta in detail.

A portion of the stock LC3-I species forms a complex with the E1-like activating enzyme Atg7 (LC3-I:Atg7 complex) and subsequently generates a conjugate (LC3-Atg7). Co-expression of GFP-Atg7 and mRFP-p62 demonstrates inclusion of GFP- Atg7 into p62 punctae (Fig. 5Aa). Inclusion of GFP-Atg7 within p62 punctae became more substantial with co-expression of GFP-Atg7, mRFP-p62 and Flag-LC3 (Fig. 5Ab) indicating an excess of LC3 (Flag-LC3) enhances the incorporation of GFP-Atg7 to p62 aggregates. In contrast, an Atg7 mutant GFP-Atg7^C572A^, which is deficient in the ability to form a conjugate with LC3, abolished sequestration when co-expressed with mRFP-p62 and Flag-LC3 (Fig. 5Ac). These observations are further detailed in Fig. 5B and 5C. It seems that conjugation of LC3 with Atg7 is required for inclusion of Atg7 into p62 puncta. However, Flag-LC3^A120^, a mutant LC3 which cannot form a conjugate with Atg7, also enhanced GFP-Atg7 sequestration into p62 aggregates (Fig. 5D). This colocalization was abolished by the co-expression of GFP-Atg7^C572A^/mRFP-p62/Flag-LC3^A120^ (Fig. 5E).

Why was the GFP-Atg7^C572A^ mutant not sequestered into p62 aggregates? To answer this question, we performed Immunoprecipitation assays. Flag-LC3 efficiently pulled-down GFP-Atg7 (Fig. 5F, Lane 5). In sharp contrast, a negligible amount of GFP-Atg7^C572A^ was pulled-down by Flag-LC3 (Fig. 5F, Lane 6). A similar result was found with Flag-LC3^A120^ and GFP-Atg7 in a co-expression setting (Fig. 5G, Lanes 5 and 6). These results demonstrate that wild-type GFP-Atg7 and Flag-LC3 or GFP-Atg7 and Flag-LC3^A120^ form complexes within the cell despite Flag-LC3^A120^ being unable to form a conjugate with GFP-Atg7. The C572A mutation of Atg7 abolished the complex formation with the wild-type LC3 and LC3^A120^ mutant (Fig. 5F, Lane 6 and Fig. 5G, Lane 6). The inability of LC3 or LC3^A120^ to form a complex with GFP-Atg7^C572A^ causes the mutant to be absent in p62 aggregates.

Both LC3 and Atg12 conjugation systems utilize Atg7 as an E1-like activating enzyme. If Atg12 could potentially interact with p62, Atg7 would subsequently be sequestered by p62 aggregates in the same manner as LC3. To clarify this, we co-transfected mRFP-p62 and GFP-Atg12 expression plasmids into MEFwt cells. Unlike the interaction of GFP-LC3 and mRFP-p62 in MEFwt cells (Fig. 4, C), Atg12 was not sequestered within p62 aggregates (Fig. 5 H). This demonstrates that Atg12 does not interact with p62 and has no role in sequestration of Atg7 by p62 aggregates.

Altogether, these results demonstrate that p62 sequesters Atg7 within p62 protein aggregates in a LC3-dependent manner.

### Atg3 and LC3-Atg3 Conjugate are Protected from Sequestration within p62 Aggregates

Should the LC3-Atg3 conjugate be generated by interaction of Atg3 with LC3-Atg7 conjugate or LC3:Atg7 complex, then at the stage when Atg3 is complexed with LC3-Atg7 or LC3:Atg7 it could potentially be captured by p62 and included into p62 aggregates via the interaction with LC3.

**Figure 6:**
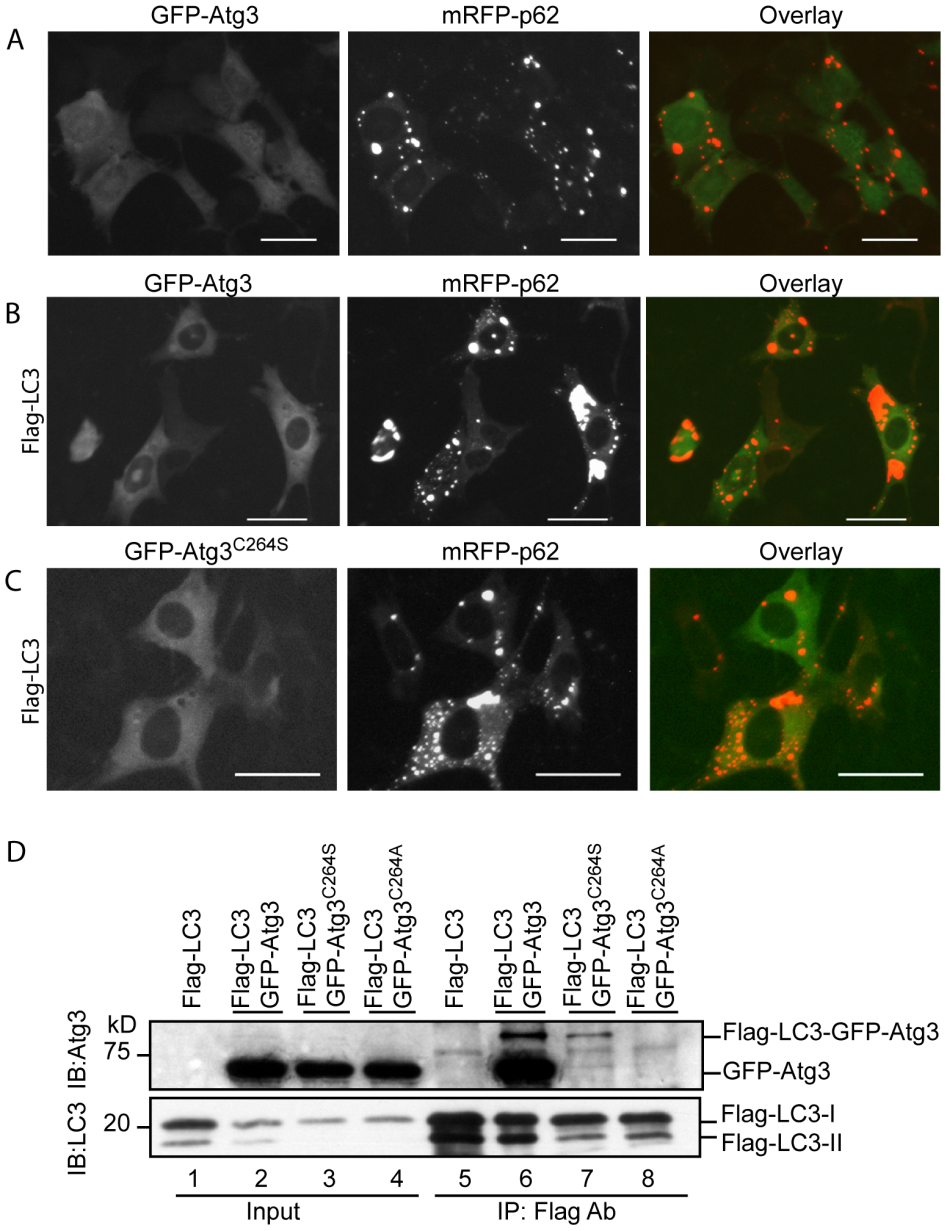
p62 aggregates do not sequester Atg3 and LC3-Atg3. GFP-Atg3 and mRFP-p62 (A) or with Flag-LC3 vectors (B) were co-transfected into MEFwt cells for 48 h. GFP-Atg3^C264S^, mRFP-p62 and Flag-LC3 vectors were co-transfected into MEFwt cells for 48 h (C). Flag-LC3, GFP-Atg3, GFP-Atg3^C264S^, or GFP-Atg3^C264A^ expression vectors were co-transfected into HEK293 cells for 48 h. Immunoprecipitation was conducted using total cell lysates. Immunoblotting was conducted as indicated. Images were recorded at 48 h post-transfection. Scale bar = 25 micron.

Co-expression of GFP-Atg3/mRFP-p62, GFP-Atg3/mRFP-p62/Flag-LC3 or GFP-Atg3^C264S^/mRFP-p62/Flag-LC3 revealed that none of the wild-type GFP-Atg3 or its mutant GFP-Atg3^C264S^ was incorporated within p62 punctae (Fig. 6A, B and C). IP assay demonstrated that Flag-LC3 formed a complex with wild-type Atg3, while it did not form a complex with Atg3^C264S^ (forming a stable O-ester bond with LC3) and Atg3^C264A^ (no bond formation with LC3) (Fig. 6D). Flag-LC3-GFP-Atg3 and Flag-LC3-GFP-Atg3^C264S^ conjugates were observed (Fig. 6D, Lanes 6 and 7). In contrast, no such larger species band was found with the GFP-Atg3^C264A^ mutant (Fig. 6D, Lane 8).

These data demonstrate that Atg3 does not interact with p62, and once bound to LC3 as an LC3:Atg3 complex or a conjugate with LC3 (LC3-Atg3), Atg3 blocks the interaction of LC3 with p62. Thus, Atg3 and LC3-Atg3 conjugate are protected from being sequestered within p62 protein aggregates.

### Atg4B, Atg3 and p62 are Mutually Excluded in the LC3 Complex

**Figure 7:**
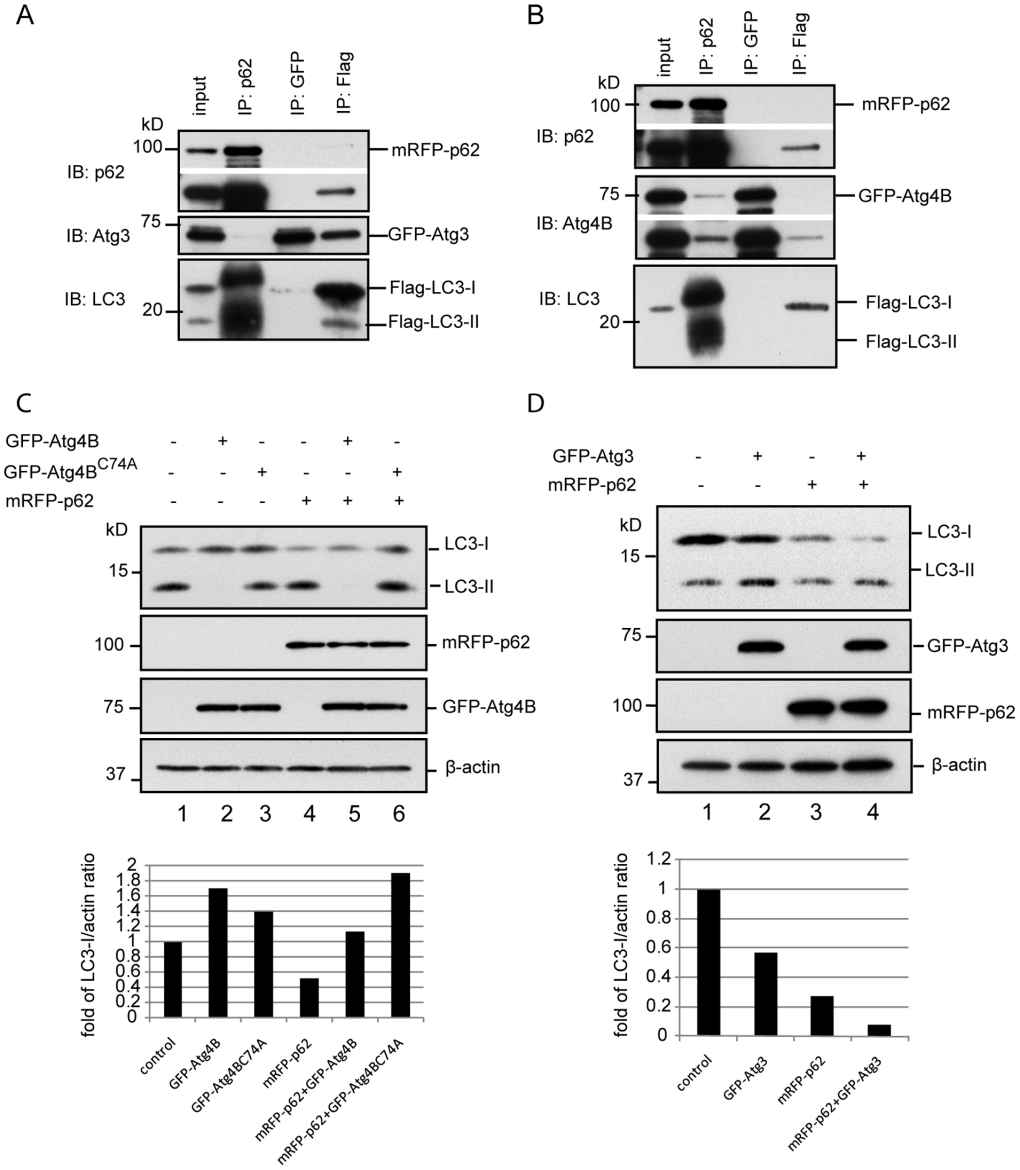
Mutual exclusion of Atg4 or Atg3 with p62 in LC3 complex. HEK293 cells were co-transfected with mRFP-p62/GFP-Atg3/Flag-LC3 or mRFP-p62/GFP-Atg4B/Flag-LC3 for 48 h. Total cell lysates were used to conduct IP using correspondent antibodies. Immunoblottings were then applied using antibodies as indicated (A and B). HEK293 cells were transfected with GFP-Atg4B or GFP-Atg4B^C74A^ alone or with mRFP-p62 for 48 h (C). HEK293 cells were transfected with GFP-Atg3 alone or with mRFP-p62 for 48 h (D). Total cell lysates were used to detect LC3 species as indicated. Relative band intensity of protein of interest was divided by correspondent band intensity of β-actin loading control. Fold of increase or decrease was then determined by setting the control sample as 1.

We have demonstrated that Atg4B and Atg3 are not included in p62 aggregates although both of them interact with LC3. In the excess of Atg4B^C74A^, we found that Flag-LC3 can pull-down a larger amount of Atg4B^C74A^ with no Atg4B^C74A^ signal revealed in mRFP-p62 aggregates (Fig. 3, E, F and G). To verify the mutual exclusion of these proteins in the LC3 complex, we did IP assays using the cell lysates from HEK293 cells. In the mRFP-p62/GFP-Atg3/Flag-LC3 co-transfection setting, we found that the pulled-down mRFP-p62 was accompanied with significant amount of Flag-LC3-I and Flag-LC3-II. No GFP-Atg3 was captured by mRFP-p62; pull-down of GFP-Atg3 did not accompany with mRFP-p62 but with a little amount of Flag-LC3-I; Again, GFP-Atg3 was pulled-down by Flag-LC3. No significant amount of mRFP-p62 was captured by Flag-LC3 (Fig. 7A). These results demonstrate that Atg3 and p62 are mutually excluded once they form a complex with LC3; only a portion of Atg3 binds to LC3, supporting Atg3 functioning as E2-like ligase, i.e., Atg3 binding to LC3 and releasing LC3 is rapid and dynamic. Similar findings were revealed in the mRFP-p62/GFP-Atg4B/Flag-LC3 co-transfection setting (Fig. 7B). Of note, GFP-Atg4B pulled-down little amount of Flag-LC3. Reversely, Flag-LC3 captured a little amount of GFP-Atg4B, supporting that Atg4B works as a cleaving enzyme, i.e., binding and processing of pro-LC3 by Atg4B are rapid and dynamic.

Together, IP assays demonstrate that p62 does not capture Atg3 and Atg4B even at an excess of LC3 condition, supporting the mutual exclusion idea when p62, Atg3 or Atg4B form a complex with LC3.

A straightforward idea for these findings would be if an excess of Atg4B or Atg3 could protect stock “LC3-I” from being sequestered into p62 aggregates. To answer this question we co-transfected mRFP-p62/GFP-Atg4B or mRFP-p62/GFP-Atg3 into HEK293 cells. As shown in Fig. 7C, GFP-Atg4B alone indeed increased LC3-I level. However, this increase in LC3-I is likely due to cleavage of LC3-II by the excess of GFP-Atg4B [22], which is also supported by little amount of LC3-II in the cells with over-expression of GFP-Atg4B (lane 2 and lane 5). However, GFP-Atg4B^C74A^ mutant indeed resulted in increase of LC3-I levels in GFP-Atg4B^C74A^ alone and GFP-Atg4B^C74A^/mRFP-p62 expression settings (Fig. 7C, lane 3 and lane 6). This result indicates that if Atg4B could tightly bind to LC3 (such as in the Atg4B^C74A^ situation), LC3 could be protected from being sequestered into p62 aggregates. However, because wild-type Atg4B’s rapid and dynamic binding and releasing LC3, it would not protect LC3 from being attacked by p62 aggregate. On the other hand, an excess of Atg4B will delipidate LC3-II to LC3-I and make LC3-I vulnerable for p62 attack (Fig. 7C, lane 5).

In the mRFP-p62/GFP-Atg3 over-expression situation, we observed a decrease in LC3-I levels and an increase in LC3-II levels (Fig. D, lane 2 and lane 4). Thus, over-expression of Atg3 actually promotes autophagy. Why is this? First, biological function of Atg3 is to exchange Atg7 in LC3-Atg7 conjugate. Because of the limitation of Atg7 in quantity, it is genetic inherency that LC3:Atg7 complex and LC3-Atg7 conjugates would not exist in a larger amount in the cell. Consequently, the quantity of Atg3 bound to LC3, LC3:Atg7 or LC3-Atg7 is limited. Thus, majority of LC3 does not bind to Atg3 and will not be protected from being attacked by the excess of p62. Second, an excess of Atg3 may enhance the exchange of LC3-Atg7 to LC3-Atg3, which eventually promotes LC3-I to LC3-II conversion (Fig. 7D, lane 4).

### p62 is Preferentially Recruited by LC3-II Under Autophagic Stimulation

As already shown in Fig. 7 A and B, over-expressed mRFP-p62 captured both Flag-LC3-I and Flag-LC3-II. Compared to input and pulled-down Flag-LC3, mRFP-p62 preferentially binds to Flag-LC3-II. It is also clear that pulled-down Flag-LC3 does not accompany with equal amount of mRFP-p62 (Fig. 7 A and B). These data support the notion that LC3 does not randomly interact with p62.

We previously reported that calcium phosphate precipitates (CPP) can induce intensive autophagic response [24]. CPP treated cells are able to generate large LC3-II positive vesicles. These LC3 decorated large vesicles with clear lumen can be easily observed under fluorescence microscopy. Using this model, we can differentiate membrane bound LC3 (LC3-II) from other LC3 species.

**Figure 8:**
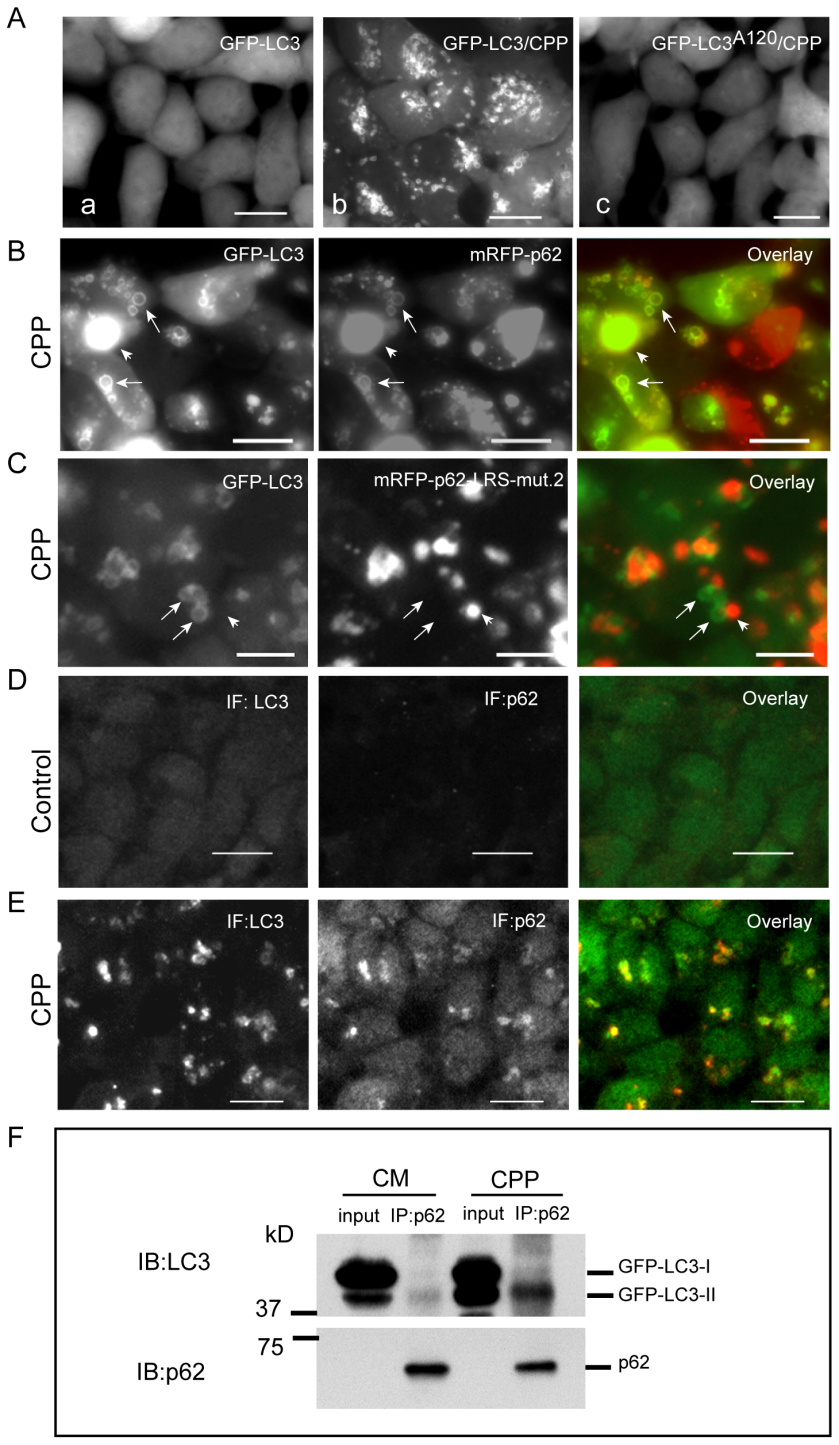
p62 preferentially binds LC3-II under autophagic stimulation. HEK293-GFP-LC3 cells were cultured in nutrient-rich media (Aa) or treated with CPP for 2 h (Ab) to induce large GFP-LC3 positive vesicles. HEK293-GFP-LC3^A120^ cells were used as a control (Ac); (B) HEK293-GFP-LC3 cells were transfected with mRFP-p62 for 24 h, and then treated with CPP for 2 h to induce large GFP-LC3/mRFP-p62 positive vesicles. (C) HEK293-GFP-LC3 cells were transfected with mRFP-p62-LRS-mut.2 for 24 h, and then treated with CPP for 2 h. HEK293-GFP-LC3 cell line was cultured in regular media (D) or treated with CPP for 2 h (E) before fixed and immunostained for the endogenous LC3 and p62. (F) HEK293-GFP-LC3 cells were cultured in regular media or treated with CPP for 2 h. Total cell lysates were used for immunoprecipitation of endogenous p62. Western blot analysis was conducted to detect p62 bound GFP-LC3 species. Arrow points to GFP-LC3 positive vesicle. Arrow head points to p62 aggregate. Scale bar = 25 micron.

Few GFP-LC3 punctae were observed in HEK293-GFP-LC3 cells under normal culture condition (Fig. 8Aa). After CPP treatment, large GFP-LC3 positive vesicles were generated (Fig. 8Ab). In contrast, HEK293-GFP-LC3^A120^ cells showed no GFP-LC3^A120^ punctae under CPP stimulation (Fig. 8Ac). This demonstrates that the wild-type GFP-LC3 but not the mutant GFP-LC3^A120^ passes through the LC3 lipidation process and targets the autophagosome membrane as the GFP-LC3-II form. As shown in Fig. 8B, GFP-LC3-II together with mRFP-p62 clearly targeted the vesicle membrane in HEK293-GFP-LC3 cells with CPP treatment (Fig. 8B, pointed by arrows). A mutant p62, which abates the p62 interaction with LC3, was found absent on the GFP-LC3 positive vesicle membranes under CPP treatment (Fig. 8C). Similar observations were confirmed by immunofluoresence staining of endogenous LC3 and p62 (Fig. 8, D and E).

To further prove the finding that LC3-II preferentially recruits p62, we performed IP assays in HEK293-GFP-LC3 cell line. Cells were cultured in complete media (CM) or treated with CPP for 2 hours. Total cell lysates were used for IP assays. Results showed that p62 pulled-down equivalent amount of GFP-LC3-II with a little amount of GFP-LC3-I in both control and CPP treated cells (Fig. 8F).

Collectively, these results demonstrate that LC3-II is responsible for the recruitment of p62 onto the vesicle membrane and p62 preferentially binds LC3-II under autophagic stimulation.

### p62 Protein Aggregates’ Effect on the Levels of Cytosolic LC3 and Atg7

**Figure 9:**
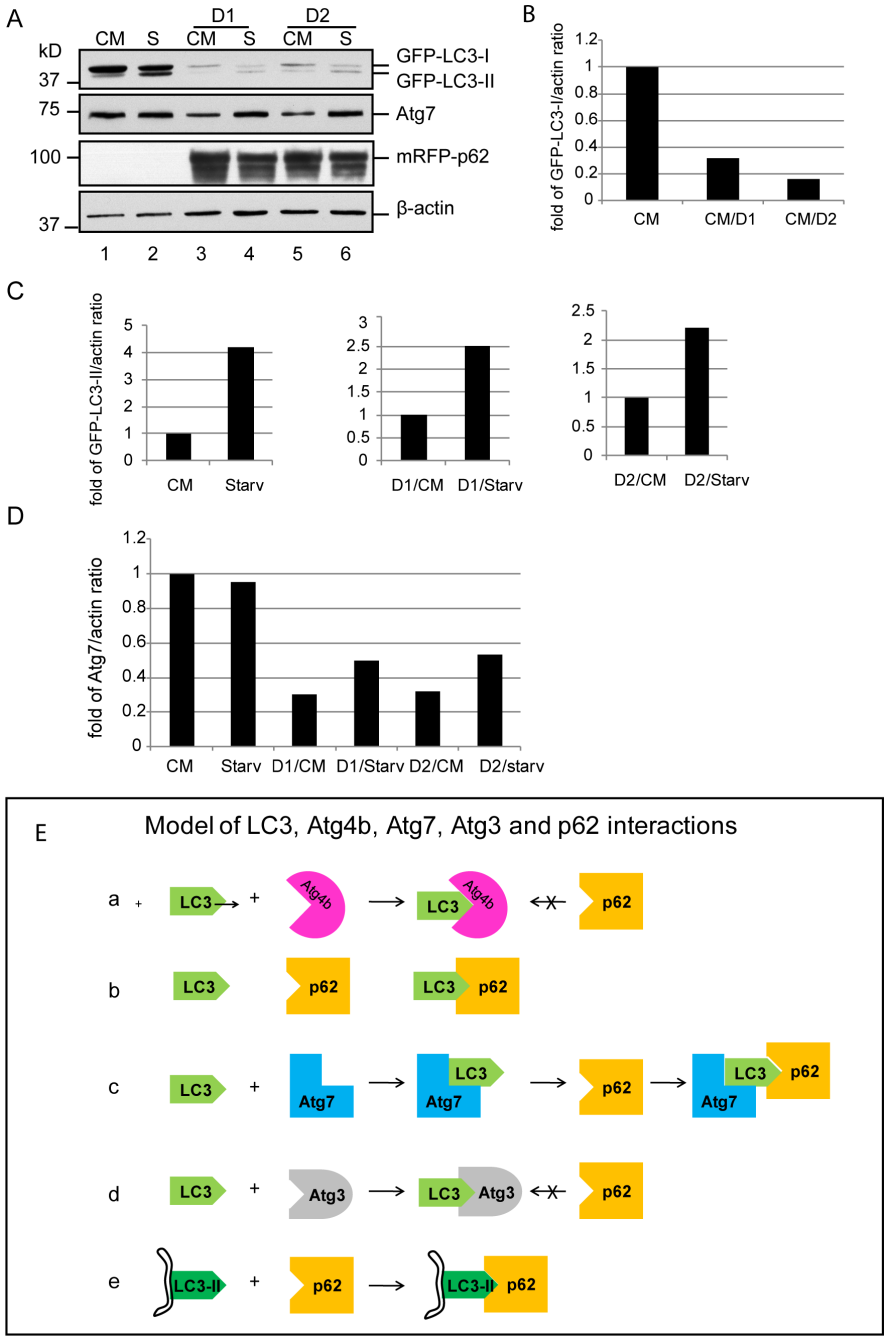
(A) p62 aggregates’ effect on cytosolic LC3 and Atg7 levels. A549 cells stably expressing GFP-LC3 were infected with Ad-mRFP-p62 at 20 MOI (D1) and 10 MOI (D2) for 48 h. Cells were then cultured in nutrient-rich media or starved in EBSS for 2 h. Total cell lysates were centrifuged at 15,000 g to separate p62 aggregates. The supernatants were used for immunoblot assays as indicated. Image J was used to quantify the related band densities using β-actin as a loading control. Relative band intensity of protein of interest was divided by the correspondent band intensity of β-actin. Fold of increase or decrease was then determined by setting the control sample as 1. CM = complete media. S = starvation. (B) Summary of p62 interaction with LC3. As Atg4B occupies or blocks the binding site for p62, there is no interaction between p62 and LC3 (a). Atg7 binds LC3 but does not block the p62 interaction domain allowing the interaction of p62 and LC3 (b). As Atg3 occupies or blocks the binding site for p62, there is no interaction between p62 and LC3(c). Membrane bound LC3-II exposes the binding site for p62 after it is converted from the LC3-Atg3 and becomes the receptor for p62.

To examine whether p62 could negatively affect LC3 lipidation, we infected A549 cells stably expressing GFP-LC3 with an adenovial vector encoding mRFP-p62. The cytosolic GFP-LC3-I was dramatically reduced in cells expressing mRFP-p62 (32 fold and 16 fold decrease in D1 and D2, respectively) (Fig. 9A, lane3 and 5, and Fig. 9B). Under starvation conditions, GFP-LC3-II increased significantly in control cells (4.2 fold increase) (Fig. 9A, lane 2, and Fig. 9C, left panel). Although starvation stimulation increased GFP-LC3-II levels in mRFP-p62 over-expression cells, they were significantly decreased (2.5 and 2.2 fold reduction for D1 and D2, respectively) (Fig. 9A, lane 4 and 6, and Fig. 9 C, middle and right panels). As expected, cytosolic Atg7 was significantly reduced in the mRFP-p62 expression cells (0.7 and 0.68 fold reduction in D1 and D2, respectively) (Fig. 9A, lane3 and 5, Fig. 9 D). Intriguingly, Atg7 was upregulated under nutrient deprived conditions (0.2 and 0.21 fold increase in D1 and D2, respectively) (Fig. 9A, lane 4 and 6, and Fig. 9 D).

In context with the previous findings in this study, these data demonstrate that the sequestration of LC3-I species by p62 aggregates significantly decreases LC3-II generation in quantity. Reduction in LC3-II production may lead to the failure to sequester p62 protein aggregates into the autophagosome. These results, on the other hand, indicate a self-protection mechanism of the cell to maintain LC3 lipidation by upregulating Atg7 expression and by preventing LC3-Atg3 species from being sequestered within p62 aggregates.

## Discussion

Our study reveals distinct p62 interactions with LC3 species in the course of LC3 lipidation. Figure 9E summarizes these findings. Our data may reflect the ability of p62 to negatively influence the LC3 lipidation process or conversely LC3 lipidation could affect p62 turnover by autophagy. We have shown that p62 interacts with LC3-I and LC3-I:Atg7 complex. These early LC3 species in LC3 lipidation could be potentially sequestered within p62 protein aggregates in pathologic conditions. On the other hand, cells may likely develop a protective mechanism to prevent key LC3 species from being sequestered into p62 aggregates by eliminating their respective interactions when pro-LC3 or LC3-I complexes with Atg4B for processing or at a particularly important stage of the LC3-Atg3 intermediate. Lastly, the preferential binding to LC3-II under autophagic stimulation favors its degradation by autophagy and minimizes any inhibitory effect p62 may have on the lipidation process. This essentially allows for a “feedforward” response when autophagy is stimulated.

There are three isoforms of Atg7 mRNA in the mammalian cell (gene bank, AC022001, AC083855, AF094516, AL1222075, and DA177126). This may imply Atg7 isoforms serve an evolutionary and conserved role in minimizing the effect LC3 interacting proteins such as p62 have on LC3 lipidation. Excess of Atg7 isoforms could potentially attenuate the side effect p62 has on the LC3-PE lipidation process if p62 would form protein aggregates. In addition, Atg7 is shared within the LC3 and Atg12 conjugation systems. The sequestration of Atg12 within p62 aggregates was not observed (Fig. 5H). In a physiologic condition, Atg7 may form a complex with a portion of LC3-I species for LC3-Atg7 conjugate formation and a portion of Atg12 for Atg12-Atg7 conjugate formation. For these reasons, Atg7 would not be fully consumed by p62 protein aggregates. Intriguingly, Atg7 was upregulated under starvation conditions in the situation when p62 aggregates. This finding needs a further investigation. We hypothesize that in certain conditions where Atg7 may be down-regulated such as in the context of neural or other senescent cells, p62-mediated protein aggregates could negatively affect the LC3-PE lipidation process and attenuate autophagy and subsequently trigger the development of a diseased state.

In contrast to Atg7, Atg3 has only one encoding mRNA with no putative Kozak sequence flanking the start codon region of its mRNA, suggesting that Atg3 is a low level expression protein. In context with the findings of p62 binding LC3-I:Atg7 complex, should Atg3 interact with only Atg7 in LC3-Atg7 intermediate or LC3:Atg7 complex to accomplish LC3-Atg3 conjugation [25], p62 would not block Atg3 interaction with Atg7. However, we did not observe any form of Atg3 included in the p62 aggregates. This may represent a physiologic mechanism to protect LC3:Atg3 complex or LC3-Atg3 intermediate from being subsequently trapped within p62 aggregates and impair autophagy.

Although p62, Atg4B and Atg3 interact with LC3 species, it seems that they are mutually excluded from each other once they bind to LC3 (Fig. 7 A and B). We hypothesize that Atg4B and Atg3 may share a similar mechanism to block p62 once they form a complex with LC3. This likely occurs via Atg4B or Atg3 occupying the same interaction domain on the LC3 molecule.

Altogether, we propose a working model for the involvement of p62 in autophagy based on ours and others’ findings. As the LC3-Atg3 intermediate interacts with the Atg16L1:Atg5-Atg12 complex on the phagophore membrane (at this stage, p62 does not bind to LC3-Atg3 intermediate), Atg3 in the intermediate exchanges with PE on the phagophore guided by Atg16L1:Atg5–Atg12 complex to generate LC3-II. As Atg3 releases from the LC3-Atg3 intermediate, LC3-II on phagophore membrane exposes its p62 interaction site once occupied by Atg3. These LC3-IIs on the phagophore membrane are energetically ready to receive p62. Via its interaction with LC3-II, p62 is subsequently sequestered into the autophagosome. Similarly, p62 protein aggregates could be sequestered into the autophagosome by interaction with LC3-II on the phagophore membrane. In such a model, p62 exposed on the surface of aggregates can either interact with LC3-II (free of p62) or with the p62 on the phagophore membrane (as p62 bound LC3-II) via its PB domain (for p62 oligomerization) and eventually drive the phagophore membrane to sequester the p62 aggregate. However, in a condition of p62 mediated protein aggregation p62 could negatively affect LC3 lipidation process by sequestration of LC3-I and Atg7. Thus, p62 aggregates fail to be delivered into the autophagosome for degradation. Impaired autophagy may lead to p62-mediated protein aggregate disease development.
